# Complicated Urinary Tract Tuberculosis in a 13-Year-Old Adolescent with Chronic Kidney Disease and Antituberculous Drug-Induced Hepatotoxicity

**DOI:** 10.1155/2019/7370150

**Published:** 2019-10-23

**Authors:** Heda Melinda Nataprawira, Adhitya Agung Pratama, Ahmedz Widiasta, Jupiter Sibarani, Dany Hilmanto, Nanan Sekarwana, Dedi Rachmadi

**Affiliations:** ^1^Respirology Division, Department of Child Health, Faculty of Medicine, Universitas Padjadjaran, Hasan Sadikin General Hospital, Pasteur 38, Bandung, West Java 40161, Indonesia; ^2^Nephrology Division, Department of Child Health, Faculty of Medicine, Universitas Padjadjaran, Hasan Sadikin General Hospital, Pasteur 38, Bandung, West Java 40161, Indonesia; ^3^Urology Department, Department of Child Health, Faculty of Medicine, Universitas Padjadjaran, Hasan Sadikin General Hospital, Pasteur 38, Bandung, West Java 40161, Indonesia

## Abstract

Urinary tract tuberculosis (TB) is a rare extrapulmonary manifestation of TB in children. The disease is potentially underdiagnosed because it clinically resembles other urinary tract infections. A 13-year-old adolescent girl presented with pain, difficulty in micturition, and gross hematuria for almost two years before admission, and she had left flank pain since one year ago and significant loss of body weight during the illness. The close TB contact was her grandmother who was on TB treatment. Acid-fast bacilli yielded positive result, Mantoux test was positive (17 mm), urine GeneXpert MTB/Rif was positive; tuberculoma was identified on kidney histopathology, and a diuretic renogram revealed an uncorrected glomerular filtration rate (GFR) of the right and left kidney to be 32.5 mL/min/1.73 m^2^ and 5 mL/min/1.73 m^2^, respectively. During the treatment, oral anti-TB drug-induced hepatotoxicity (ADIH) occurred to the patient. This problem was solved with management according to the British Thoracic Society (BTS) guidelines. Screening TB in children is very important for a better outcome. If children complain of some complicated urinary tract infection, TB should be suspected. Optimaly treating children with urinary tract TB exagerrated with ADIH and CKD is very challenging.

## 1. Introduction

Tuberculosis (TB) is still a burden of social health in the world according to the World Health Organization (WHO) report. Indonesia is in the second position on the list of TB patients globally. The World Health Organization Global Report 2017 declared that extrapulmonary TB accounted for 15% of all TB cases in the world. Urinary tract TB is a rare case in children, represents less than 5% of pediatric extrapulmonary TB [1, 2]. Tuberculosis in younger children is the most challenging, especially in countries with limited resources and high endemicity like ours. The urinary tract TB is potentially underdiagnosed because of the nonspecific urinary tract symptoms such as urethral stricture, contracted bladder, and even chronic kidney disease [3–6]. Urinary tract TB has a wide spectrum of symptoms, but the most common is pain on micturition and hematuria [2]. Gold standard for the diagnosis of urinary tract TB is established through the isolation and culture of *Mycobacterium tuberculosis* [1]. Early diagnosis of urinary tract TB could prevent the sequelae such as kidney impairment [1, 3–5]. In this report, we also evaluated a stunted school girl who presented with complain in micturition.

## 2. Case Report

A 13-year-old girl from a remote village of Bandung presented with urology emergency to our hospital with gross hematuria and difficult and painful micturition since 2 years before admission. She had flank pain as well since a year ago. There was no history of cough, night sweats, malaise, or swelling of abdomen or extremities. The patient had lost her weight for about 2 kg in a month. There was no history of TB disease previously, but her grandmother was diagnosed as having pulmonary TB and was on TB treatment.

Physical examination showed undernourishment, body weight 27 kg (–2 to –3SD), and height 137 cm (–2 to –3SD). On examination, her general appearance was moderately ill and blood pressure was within the normal limit and had no fever. There was pain on the left flank. Urine microscopy showed leukocyturia, hematuria, and positive acid-fast bacilli, and *M. tuberculosis* was detected in rapid-test molecular test (gene Xpert). Laboratory findings showed serum creatinine 0.80 mg/dL, serum urea 12.0 mg/dL, serum sodium 142 mEq/L, serum potassium 4.5 mEq/L, serum calcium 5.61 mg/dL, Hb 10.6 g/dL, hematocrit 33.9%, WBC 11,800/mm3, platelet count 663,000/mm3, differential count 0/2/0/73/19/6, and quantitative CRP 1.2 mg/dL, and anti-HIV, anti-HCV, and HbsAg were negative. Ultrasound of kidney-ureter-bladder showed bilateral kidney pelvocaliectasis, and diuretic renogram revealed an important difference in renal function, i.e., the uncorrected glomerular filtration rate (GFR) of the right kidney and the left kidney was 32.5 mL/min/1.73 m^2^ and 5 mL/min/1.73 m^2^, respectively. Chest radiograph showed active pulmonary tuberculosis; Mantoux test showed positive result (an induration of 17 mm). Based on those findings, we diagnosed complicated urinary tract infection, left ureteral stenosis, bilateral hydronephrosis, and stage 3 chronic kidney disease (CKD). Left kidney nephrectomy and bladder augmentation were performed by a pediatric urologist, and then the anatomopathological result was pyelonephritis tuberculosis (tuberculoma).

**Figure 1:**
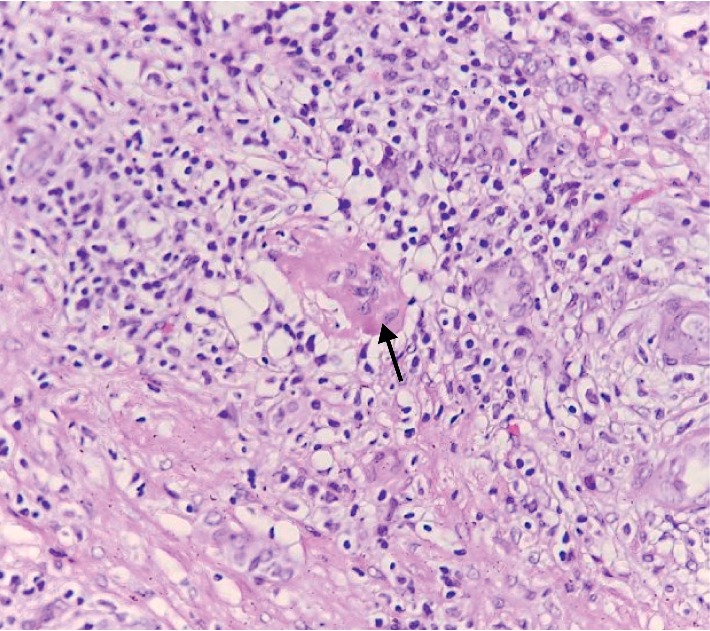
Photomicrograph (H and E x400) showing a multinucleated giant cell that is characteristic of tuberculoma.

The patient was treated with four antituberculous drugs in the intensive phase which she had completed and then continued with two antituberculous drugs in the continuation phase. She experienced antituberculous drug-induced hepatotoxicity (ADIH) after two months of antitubercular treatment (ATT). We diagnosed the patient as complicated urinary tract TB, stage 3 chronic kidney disease (CKD), and ADIH. The patient was given ethambutol and streptomycin with an adjusting dose according to GFR and was given calcium carbonate, folic acid, and vitamin D orally. The child gained 2.5 kilograms during this time period, and her condition improved significantly (Figure 1).

The patient was discharged in good condition, without any hypertension and other chronic kidney disease symptoms; the GFR was 29.5 mL/min/1.73 m^2^ and bladder capacity was 250–300 mL after having bladder augmentation. Her quality of life was gradually getting better according to the pediatric symptoms checklist for quality of life (Peds-QL) of patient age 13–18 years old, and she can perform some daily activity well without any physical and psychological disturbances.

## 3. Discussion

Complicated urinary tract TB is a rare disease with unusual symptoms in children; our patient's complaints were painful and difficult micturition, gross hematuria, left flank pain, and loss of body weight, with a close tuberculosis contact. The complaints according to other reports were abdominal pain and constipation, with hard-elastic left abdomen. This patient had felt symptoms of TB since two years, and according to the time table of Wallgren, urinary tract TB happens after five years of TB infection [7, 8]. This patient had symptoms that were clinically similar with other complicated urinary tract infections, supported by left ureteral stenosis from ultrasound examination, strongly suggestive of a congenital anomaly of the kidney and urinary tract (CAKUT). The CAKUT usually becomes progressive chronic kidney disease within a decade [9, 10].

The diagnosis was suggested as complication due to *E. coli* because of her age. The urinary culture result was *Enterobacter cloacae* with the bacterial count more than 10^5^ cfu/hpf. The left kidney's glomerular filtration rate (GFR) from the renogram was very low; then, we decided to perform nephrectomy of the left kidney and analyze the histopathology of the kidney, and the result was tuberculoma. We performed urine GeneXpert, and the result was positive, which means that the bladder was infected by *Mycobacterium tuberculosis*. Our suggestion is that TB infection had began from the bladder and then ascended to the ureter and the kidney. The bladder inflammation caused a change in bladder pressure leading a vesicoureteral reflux. This process had happened for a long time before hydronephrosis occurred.

This disease represents less than 5% of pediatric extrapulmonary tuberculosis. The disease is a late reactivation or complication of pulmonary tuberculosis, mostly present in 20–40 years [1]. Research conducted by Wise et al. reported the mean age of tuberculosis patients is 40.7 years with a range of 5–90 years. The long latent period about 4–10 years of age causes urinary tract involvement rarely at the age of the children. Nerli et al. conducted a study in India of 17 children diagnosed with urinary tract tuberculosis, and the age of the 17 children was in the range of 7–13 years [3–5, 7, 11–13].

Arora et al. reported three case reports in India, one of them with symptoms and signs which resemble our patient. The patient was a 13-year-old boy, presented with fever, hematuria, burning micturition, massively proteinuria, and cervical lymphadenopathy. The Mantoux test was strongly positive. Chest radiograph presented right hilar lymphadenopathy, and ultrasonography showed evidence of acute pyelonephritis. The fine-needle aspiration cytology (FNAC) result established tuberculous lymphadenitis, and he responded to ATT. Follow-up urinalysis and ultrasound were normal [13, 14].

This case has an earlier age than the previous reports, and the symptoms resemble with other complicated urinary tract infection, so it was very difficult to make urinary tract tuberculosis diagnosis without histopathologic examination.

Approximately, 25% of tuberculosis cases might affect extrapulmonary organs through hematogenous and lymphatic spread [1]. Urinary tract tuberculosis is one of the rare manifestations of *Mycobacterium tuberculosis* (MTB) infections [1]. The incidence rate is less than 5% of all tuberculosis manifestations [3]. The common symptoms are pain on the abdomen, waist, and lower back or difficult micturition [3]. Systemic symptoms such as fever, weight loss, and decreased appetite are rarely complained [3]. The symptom in these patients was difficult and painful micturition that has been felt since two years before admission [15–19].

Urinalysis might show hematuria and pyuria with sterile urine culture results. Acid-fast bacilli (AFB) could also be detected in 80–90% of cases of urinary tract tuberculosis. Examination of the urine culture usually takes a long time, which is about 6–8 weeks, and the probability of false-negative results is 10–20% [19].

Urinary tract tuberculosis resulting from hematogenous spread begins with the formation of primary tuberculosis focus [1, 3, 4, 20]. *Mycobacterium tuberculosis* has a high resistance to the destruction process, so that the bacteria duplicate in the macrophages and then carry into the lymphatic and hematogenous flow by bringing macrophages that contain the bacteria throughout the body until it ends in the kidney [1]. Selective immunity will form and inhibit multiplication of bacteria and retain it in the macrophages by forming microscopic granulomas [21–26].

Granulomas will eradicate or trap the bacteria for several years in the good immunity state. Differently, with the immunocompromised state, they might reactivate or reinfect, and one or two tubercles might became enlarged after several years of inactivity [1]. This inactive period may last from 5 to 40 years [1]. *Mycobacterium tuberculosis* that is locked in the periglomerular capillaries of the kidney developed into macroscopic granuloma [1]. Some granulomas then became enlarged and united, with the inner tuberculosis bacillus filling the nephron and reach the small part of the loop of Henle, forming the focus of infection within the kidney pyramid [1]. These papillary lesions merge and form the “ulcero-cavernous” lesion that ends in the pelvical system [1, 3, 4]. Extensive papillary necrosis develops and forms a cavity and then destroys the kidney parenchyme [1]. At an advanced stage, the disease induces scars on the kidney cortex resulting in infundibular and pelvicoureteral junction stricture which would result in decreased function because of the extensive calcification that involves the entire kidney [1, 3, 4]. The young age and malnourished condition of our patient are predisposing factors for the reactivation for tuberculosis [6, 20, 21, 24].

Acid-fast bacilli (AFB) examination in the urine with Ziehl–Neelsen staining should be checked 3 times in a row, but its sensitivity is very low (40%) although its specificity is high (96.7%), with the positive result only when 5,000–10,000 bacteria/mL urine is present. In these patients, urinalysis yielded positive results on the first examination. Sample culture is a gold standard for TB diagnosis with a high sensitivity and specificity, 94.3% and 85.7%, respectively. The culture yields positive results when there is ≥10 bacteria/mL urine. These patients were subjected to culture examination without growth of microorganisms [19]. The positive paraclinical finding in this patient was kidney abnormalities revealed on ultrasound and intravenous pyelography as bilateral pelvocaliectasis, contracted bladder, and left ureteral stenosis which are the common changes reported in urinary tract tuberculosis. Pathology anatomical findings identified tubercle formation consisting of caseating necrosis, epitheloid proliferation, and multinucleated giant cells (datia langhans cells). Our suggestion is that the TB infection began and made some inflammatory process from bladder then ascended to the ureter and the kidney.

Treatment of urinary tract tuberculosis has two options: medical management and operative procedure. Tuberculosis of the kidneys is a severe tuberculosis of the weight category, and the management of anti-TB treatment is included in category I, with at least 4 kinds of drugs in the first 2 months isoniazid (H), rifampicin (R), pyrazinamide (Z), and ethambutol (E) or (2HRZE), followed by 2 kinds of drugs (4HR). Treatment for 6 months is recommended for tuberculosis involving all extrapulmonary organs, except those involving meninges requiring treatment for 9–12 months. Extension of the treatment should also be considered for extrapulmonary slow response TB patients [6, 9, 27, 28].

If patients had impaired kidney function, then the safe antituberculous options are isoniazide, rifampicin, and pyrazinamide. These drugs may be administered in normal doses because they are eliminated in the bile, and they are not excreted in the kidney. Streptomycin, ethambutol, and aminoglycosides are nephrotoxic; however, streptomycin and ethambutol could still be administered by adjusting the dose with the glomerular filtration rate (GFR) [21].

Most of the first-line ATT drugs are isoniazid, rifampicin, and pyrazinamide, which have a high incidence rate of hepatotoxicity. Treatment is needed to eliminate tuberculosis, but this action will further increase the potential for toxicity to the liver. When ADIH occurs in the patient, it would reduce the efficacy of the first-line drugs, thereby lengthening the duration of the treatment and increasing the risk of treatment failure [23]. Treating TB with kidney impairment (CKD) and liver disease (ADIH) is very challenging. The patient had a difficult condition because she could not be treated adequately as she had ADIH. We change the ATT with ethambutol and streptomycin with dose adjusted with her GFR. Treatment of ADIH is immediate cessation of antituberculous drugs and supportive treatment. Earlier, children receiving antituberculosis treatment should be evaluated to assess treatment success, adherence, and monitoring of adverse drug effects. When there was an increase in the transaminase levels ≥2 times the normal value, aspartate serum transaminase (AST) monitoring is required every week for 2 weeks and then every 2 weeks until it returns to normal [16, 20, 24].

Several countries have their own guidance on dealing with ADIH diseases, such as ATS, the Centers for Disease Control (CDC), and BTS, that issued adult guidelines for monitoring and evaluation of treatments on ADIH, such as liver function examination during antituberculosis administration, diagnosis, and how antituberculosis drug reintroduce to ADIH. In order to find the appropriate monitoring schedule for ADIH patients, they conducted a study and concluded the importance of liver function audit data prior to antituberculosis treatment [20, 23–25].

Beside the medical management, we also performed surgical management. We performed nephrectomy of the left kidney and bladder augmentation because bladder TB usually results in patchy cystitis due to inflammation of the uroepithelium by the tubercle bacillus. Resultant granulomatous inflammation, caseation necrosis, and final healing by fibrosis may lead to marked contracture of the urinary bladder within a year. There are two types of lesions in the tubercular bladder: One, (the most common form) when the bladder has reduced its capacity about 150–200 mL. The other type is structural bladder contracture wherein the urinary bladder has permanently lost its capacity and has little or no value as a urinary reservoir [29]. The ileum provides an excellent pouch to enlarge the bladder capacity when only half of the bladder is planned to be removed [30].

Good collaboration between medical and surgical treatment delivers better quality of life.
